# A Novel Approach for Combating *Klebsiella pneumoniae* Biofilm Using Histidine Functionalized Silver Nanoparticles

**DOI:** 10.3389/fmicb.2017.01104

**Published:** 2017-06-16

**Authors:** Sanjay Chhibber, Vijay S. Gondil, Samrita Sharma, Munish Kumar, Nishima Wangoo, Rohit K. Sharma

**Affiliations:** ^1^Department of Microbiology, Basic Medical Sciences, Panjab UniversityChandigarh, India; ^2^Department of Chemistry, Panjab UniversityChandigarh, India; ^3^Department of Applied Sciences, University Institute of Engineering and Technology, Panjab UniversityChandigarh, India

**Keywords:** biofilm, antibacterial agents, antibiofilm agents, antibacterial nanoparticles, drug resistance, bacterial

## Abstract

Treating pathogens is becoming challenging because of multidrug resistance and availability of limited alternative therapies which has further confounded this problem. The situation becomes more alarming when multidrug resistant pathogens form a 3D structure known as biofilm. Biofilms are formed in most of the infections especially in chronic infections where it is difficult to eradicate them by conventional antibiotic therapy. Chemically synthesized nanoparticles are known to have antibiofilm activity but in the present study, an attempt was made to use amino acid functionalized silver nanoparticles alone and in combination with gentamicin to eradicate *Klebsiella pneumoniae* biofilm. Amino acid functionalized silver nanoparticles were not only able to disrupt biofilm *in vitro* but also led to the lowering of gentamicin dose when used in combination. To the best of our knowledge, this is the first study demonstrating the application of amino acid functionalized silver nanoparticles in the eradication of young and old *K. pneumoniae* biofilm.

## Introduction

Multidrug resistant bacteria are responsible for a range of serious hospital acquired (nosocomial) and community acquired infections which are difficult to treat with available antibiotics. The issue of multidrug resistance is further complicated when unicellular organisms come together to form a community that is attached to a solid surface in the form of biofilm. Epidemiological studies have shown that biofilm formation is associated with most of the human infections (Römling and Balsalobre, 2012), about 60–80% bacterial infections are linked to biofilm formation (Black and Costerton, 2010; Fuente-Núñez et al., 2014). This further complicates the treatment regimes as microorganisms growing in a biofilm are highly resistant to antibiotics because of their attachment to solid surface, presence of extracellular polysaccharide and proteins in their matrix. *Klebsiella pneumoniae* is one of the major pathogens associated with hospital and community acquired infections such as pneumonia, urinary tract infection (UTI), burn wounds, septicemia and pyogenic liver abscess (Podschun and Ullmann, 1998; Spagnolo et al., 2014). Previous *in vitro* studies have shown that *K. pneumoniae* isolated from urine samples, sputum and blood of patients are capable of forming biofilm (Yang and Zhang, 2008; Niveditha et al., 2012). This can be related to its placement among ‘ESKAPE’ pathogens (*Enterococcus faecium, Staphylococcus aureus, K. pneumoniae, Acinetobacter baumannii, Pseudomonas aeruginosa,* and *Enterobacter* species) (Pendleton et al., 2013). Alternative therapies such as the use of phytochemicals, probiotics, bacteriophage therapy, metal salts have been found promising in case of infections caused with drug resistant strains (Chhibber et al., 2008; Gonchar et al., 2013; Sowjanya and Sridhar, 2015; Chopra et al., 2016).

Silver salts are known to be highly toxic to microorganisms as they show broad spectrum of antimicrobial activity for both Gram positive and Gram negative organisms (Jung et al., 2008). The silver coated milk bottles were used to inhibit bacterial growth and silver nitrate solution was administered to prevent conjunctivitis in neonates (Crede, 1881). Silver sulfadiazine creams have long been used for the prevention of bacterial growth in burn wound patients (Moyer et al., 1965). Silver, generally used in the form of nitrate salt, has low penetration power due to its large size. Therefore, silver nanoparticles, as an alternative to silver salts has been proposed recently due to their higher surface to volume ratio (Agnihotri et al., 2014). This provides a better surface area to which a microbe is exposed, making the treatment faster and better as compared to its salt form (Singh et al., 2010). Chemically synthesized silver nanoparticles are known to have potent antimicrobial and antibiofilm activity (Kim et al., 2007; Barapatre et al., 2016). However, to the best of our knowledge, no reports are available in which antimicrobial and antibiofilm potential has been evaluated using amino acid functionalized silver nanoparticles. Broad antibacterial activity of histidine functionalized silver nanoparticles was compared with other amino acid functionalized silver nanoparticles in a preliminary study conducted in our laboratory. The activity of histidine functionalized silver nanoparticles was found to be maximum among all other amino acid functionalized silver nanoparticles (Kumar et al., manuscript communicated). Therefore, keeping this in mind, in the present study, histidine functionalized silver nanoparticles were used for the eradication of *K. pneumoniae* biofilm *in vitro.*

## Materials and Methods

### Bacterial Strain and Nanoparticles

Standard strain of *K. pneumoniae* B5055 (01:K2) obtained from Dr. Mathia Trautmann, University of Ulm, Germany was used. Various amino acid functionalized AgNPs were synthesized and characterized by UV spectroscopy, NMR spectroscopy, TEM and FTIR spectroscopy for their physical and chemical characteristics (Kumar et al., manuscript communicated). Antimicrobial potential of all these different types of nanoparticles were checked against *Escherichia coli, P. aeruginosa,* Methicillin-resistant *Staphylococcus aureus* (MRSA) and *K. pneumoniae.* Out of all these, histidine functionalized silver nanoparticles (H-AgNPs) were used in further experiments because of its broad and highest antimicrobial activity against the tested organisms (Kumar et al., manuscript communicated).

### Congo Red Agar Assay

Congo red agar assay (CRA) was performed to evaluate the role of (H-AgNPs) on biofilm formation by *K. pneumoniae*. CRA was performed according to the method given by Freeman et al. (1989). Congo red agar was prepared with brain heart infusion broth 37 g/L, sucrose 50 g/L, agar powder 20 g/L and Congo red indicator 8%. All the ingredients were added to two different flasks, each containing 20 ml of distilled water and autoclaved at 121°C, 15 pounds for 15 min. From one flask, media was cooled, poured aseptically in sterile glass petriplate, labeled as control. In another flask, 0.9 mg/ml (MIC) nanoparticles was added, poured aseptically in a sterile glass petriplate and allowed to solidify, labeled as test plate. After 24 h, both test as well as control plates were streaked with *K. pneumoniae* and incubated overnight at 37°C. Next day, both the plates were compared for the colony color. Black colonies with a dry crystalline uniformity indicate slime production (EPS) by *K. pneumoniae*, which is a characteristic constituent of biofilm matrix.

### Establishment and Processing of *K. pneumoniae* Biofilm in a 96-Well Microtiter Plate

*K. pneumoniae* biofilm was established in 96-well microtiter plate up to 7 days according to the method described already reported (Bedi et al., 2009). One hundred microliter each of nutrient broth and bacterial culture (OD_600_ = 0.3), which is equivalent to 10^8^ CFU/ml of *K. pneumoniae,* were added to the wells of microtiter plate and incubated at 37°C overnight. In each experiment, wells containing sterile nutrient broth were used as sterility control. After each day (18–24 h), planktonic bacteria were removed and a set of two wells (corresponding to each day) were washed gently three times with PBS (pH 7.4). Adherent biofilms were scraped from wells, suspended in PBS (pH 7.4) and vortexed. Microbial load of biofilm was enumerated by viable cell counting (quantitative method) and crystal violet staining (semi-quantitative method). For viable cell counting appropriate dilutions were made and plated on Macconkey’s agar and viable count estimated after overnight incubation at 37°C. For crystal violet staining, duplicate wells were stained with 0.1% of crystal violet stain for 10 min, gently washed with 200 μl fresh normal saline and then destained with 95% of ethanol. The content from wells was transferred to flat bottom plate and analyzed on ELISA reader (BIO RAD) at 595 nm. In rest of the wells, spent medium was replaced with fresh sterile nutrient broth and microtiter plate again incubated at 37°C overnight. This procedure was sustained up to seventh day of experiment.

### Effect of H-AgNPs on *K. pneumoniae* Biofilm

To determine the efficacy of H-AgNPs on *K. pneumoniae* biofilm, 200 μl (1.8 mg/ml) of H-AgNPs was added to selected wells and in case of control well 200 μl autoclaved water was added on each day up to 7 days. Microtiter plates were reincubated for 6 h at 37°C. Only selected wells in each plate were processed for viable count and CV staining where as in the rest of wells the media was replaced and plates incubated further upto 7 days. Biofilm disruption was recorded in the form of bacterial count and cell staining as compared to control wells.

### FESEM and Compositional (EDS) Analysis of Biofilm

Biofilm on glass cover slip was grown on air-liquid interface using batch culture model (Hughes et al., 1998). Sterile 24-well microtiter plate was opened under aseptic conditions, 500 μl of nutrient broth, 500 μl of bacterial culture of *K. pneumoniae* (0.3 OD at 600 nm) and a sterile cover slip was added to each well of the plate. Last well, was used as sterility control by adding 1000 μl of nutrient broth to the well. Microtiter plate was incubated at 37°C and media was replaced with fresh media each day up to 7 days. To evaluate the efficacy of H-AgNPs on the integrity of *K. pneumoniae* biofilm, 1.8 mg of H-AgNPs were added to the selected wells and in case of control well, 200 μl autoclaved water was added on each day up to 7 days. The biofilm on fifth day (which is the peak day of biofilm formation) was subjected to FESEM (Hitachi SU8010) and energy dispersive X ray spectroscopy (Brukner XFlash6130) in order to evaluate the structure as well as elemental composition of biofilm.

### Synergistic Effect of H-AgNPs and Gentamicin on Biofilm

Fractional inhibitory concentration (FIC) index showed synergy between antibiotic gentamicin and H-AgNPs (Kumar et al., manuscript communicated). To evaluate the synergistic effect of gentamicin and H-AgNPs on *K. pneumoniae* biofilms, FIC was used. Biofilm of *K. pneumoniae* was established and on each day micro titer plate was processed under sterile conditions. Nanoparticles alone (112 μg/ml), antibiotic (gentamicin) alone (25 μg/ml) and FIC of gentamicin + H-AgNPs (25 μg + 112 μg/ml), 200 μl was added to the test well and 200 μl autoclaved water was added to the control well on each day up to 7 days. The plates were incubated for 6 h at 37°C. Only selected wells of microtiter plates were processed for viable count and crystal violet staining on each day up to 7 days. Biofilm disruption was recorded in the form of bacterial count and cell staining as compared to control wells.

### Establishment and Processing of Biofilm on Cover Slip for Confocal Laser Scanning Microscopy

Biofilm was established on coverslips as described in earlier section. After overnight incubation, media from the well was removed and cover slip gently washed with normal saline thrice to remove free bacterial cells. Desired cover slips were treated with H-AgNPs alone (112 μg/ml) or gentamicin alone (25 μg/ml) or with FIC value of gentamicin + H-AgNPs (25 μg + 112 μg/ml) at 37°C for 6 h and control sample was treated similarly with autoclaved water. The biofilm was treated simultaneously on each day up to 7 days. For confocal microscopy, samples from first, third, fifth, and seventh days were stained with fluorescent dyes (Propidium iodide red and SYTO9 slide) and observed under inverted scanning confocal laser microscope (SCLM, Nikon Ti Eclipse).

### DNA Fragmentation Assay

DNA fragmentation is a biomarker of cell apoptosis leading to nuclear DNA breakdown into multiple of oligonucleosomal size fragments. H-AgNPs were allowed to interact with *K. pneumoniae* in varying concentrations to evaluate the concentration dependent fragmentation of DNA. For DNA fragmentation assay, *K. pneumoniae* pellet was dissolved in PBS to set OD (0.5) at 600 nm and to each of the four tubes, 10 ml suspension of bacterial cells was added. In control culture, 1 ml of autoclaved water was added and in second, third, and fourth tubes, 0.45, 0.9, and 1.8 mg/ml H-AgNPs were added, respectively. Tubes were incubated for 1 h at 37°C and after incubation, DNA was isolated following established method (Ausubel et al., 1992). The optical density of treated and control samples of bacterial DNA was adjusted at 260 nm and run on agarose gel (1%) at 50 v. The samples were analyzed on gel doc to check the integrity of DNA samples.

### Cytotoxic Study on Human Monocytic Immortalized Cells THP-1

The human monocytic immortalized cells THP-1 (American Type Culture Collection, United States), were used for cytotoxic studies. The cell line was cultured in RPMI 1640 containing, 2 mM L-glutamine supplemented with 10% FBS. The media was supplemented with 1% penicillin, streptomycin, and amphotericin. Each generic cell culture protocol consisted of growing cells in an incubator at 37°C in presence of 5% CO_2_ in 150 cm^2^ flasks. This was followed by replacing media every 2–3 days and passaging before confluence by disassociation with trypsin, washing and seeding new flasks or treatment wells. As a suspension cell line, THP-1 cells were passed directly into fresh medium.

### Cell Dosing and Controls

For exposure to H-AgNPs, 5 × 10^5^ cells/ml were used in all the experiments. THP-1 cells were seeded into 96-well plates and allowed to recover, attach and proliferate for 24 h at 37°C. THP-1 cells were differentiated into macrophages by culturing in medium supplemented with 200 nM PMA (phorbolmyristate acetate) for 24 h in 96-well plates. The media was then replaced with fresh media without PMA. Then, the cell culture media in all the cell types was replaced with 200 μl of respective fresh media containing different concentrations of 10, 25, 50, 100, and 200 μg/ml H-AgNPs. The cells were washed with serum free media and then trypsinized for 5 min to detach the cells from the 96-well plates. The cell suspension was centrifuged at 500 ×*g* for 10 min and washed with PBS. The cell suspension was assessed for cellular viability using trypan blue staining assay (VWR, Bellefonte, PA, United States). Percent viable cells were calculated accordingly as: (%) viable cells = total number of viable cells per ml of aliquot/total number of cells per ml of aliquot × 100.

## Results

### Congo Red Agar Assay

**FIGURE 1:**
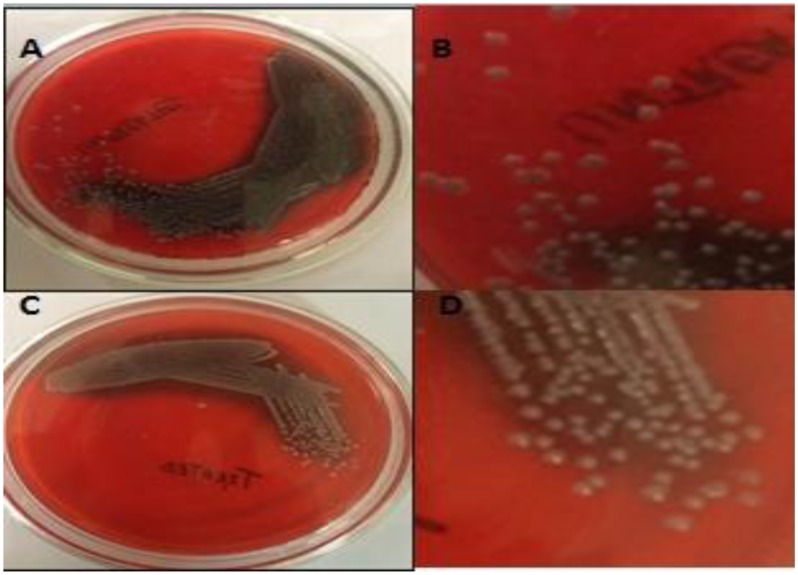
**(A,B)** Are representative pictures of Congo red agar plates in which blackening was observed confirming the EPS production by *K. pneumoniae*. **(C,D)** Show less black colonies on agar plates containing 0.9 mg per ml of H-AgNPs, decreased EPS production by *K. pneumoniae.*

Congo red agar assay showed that in the presence of H-AgNPs, EPS production was inhibited (C, **Figure 1**), as visual examination of separated colonies (D) showed less blacking of colonies. On the contrary, dark black crystalline colonies on control plate confirmed EPS production by *K. pneumoniae*, a major constituent of biofilm (**Figure 1**).

### Kinetics of Biofilm Formation

**FIGURE 2:**
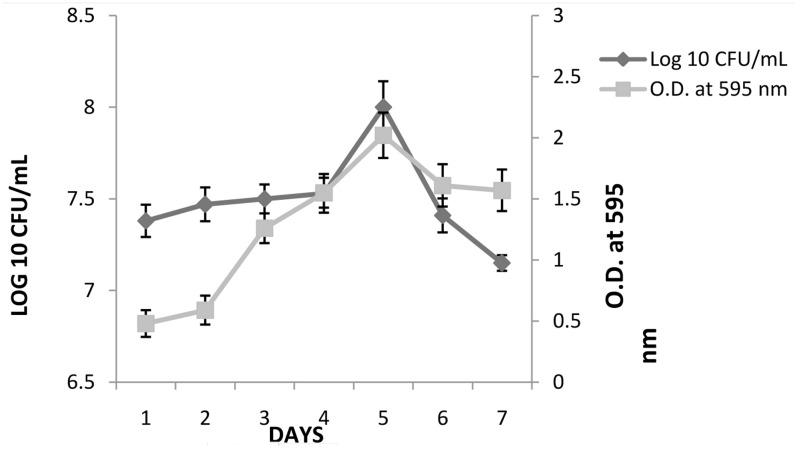
Biofilm formation by *K. pneumoniae* in the wells of 96-well micro titer plate under static conditions as determined by viable count and crystal violet (OD_595_). The experiment was performed in duplicate and repeated at least three times on different days. Error bars represent the mean ± of standard deviation.

*K. pneumoniae* was able to form biofilm on 96-well microtiter plate. The semi-quantitative and quantitative results (mean ± ; OD_595_) of biofilm formed by these strain are presented in **Figure 2**. A gradual increase in OD_595_ of *K. pneumoniae* was seen till fifth day, followed by decrease thereafter. The result of crystal violet staining (semi-quantitative estimation) confirmed the results of plate count method. On day 1, initial counts of 7.4 logs increased to 8.16 logs on fifth day. After fifth day, a gradual decrease in biofilm count was observed and 7.21 logs bacterial count was observed on seventh day.

### Effect of H-AgNPs on *K. pneumoniae* Biofilm

**FIGURE 3:**
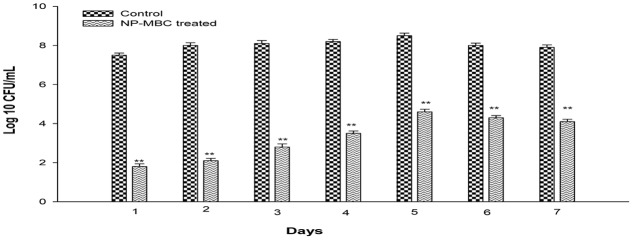
Bacterial count [Log 10 (CFU/ml)] in *K. pneumoniae* biofilm grown for different days and treated with H-AgNPs. The experiment was performed in duplicate and repeated at least three times on different days. Bars represents standard deviation and ^∗∗^ denotes *P*-value (*P* < 0.01).

Biofilm of *K. pneumoniae* was established for 7 days on 96 microtiter plates and biofilm of each day was treated with H-AgNPs at MBC concentration. Results in **Figure 3** shows that at MBC concentration, H-AgNPs led to significant (*P* < 0.01) log reduction in the bacterial count of young biofilm formed by *K. pneumoniae*. A significant gradual decrease from 8.5 to 4.6 logs in bacterial count on peak day of biofilm, i.e., fifth day was observed. Treatment with H-AgNPs also showed significant (*P* < 0.01) decrease in biofilm cell number on days 6 and 7 as well (a log reduction of 3.7 logs and 3.8 logs was observed on days 6 and 7, respectively). CV staining was also performed and showed similar results (Supplementary Data).

### FESEM and Compositional (EDS) Analysis of Biofilm

#### Morphology of Untreated Bacterial Biofilm

**FIGURE 4:**
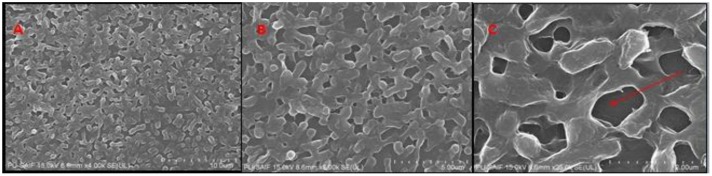
FE-SEM images of bioflim of *K. pneumoniae* on fifth day **Figure 9** **(A)**, **(B)**, and **(C)** (at × 4-25k magnification) show that bioflim forms 3D network and water channels (red arrow) along with EPS matrix.

The biofilm of *K. pneumoniae* established on cover slip was observed under FE-SEM. The results showed that biofilm had intact cells embedded in EPS matrix with 3D network and water channel (**Figure 4**).

#### Morphology of H-AgNPs Treated Bacterial Biofilm

**FIGURE 5:**
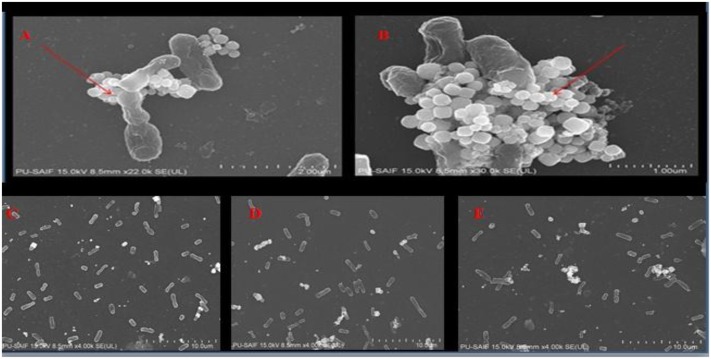
FESEM images **(A,B)** H-AgNPs were seen interacting with cells of *K. pneumoniae* (×22k-30k magnification at 15 kV). **(C–E)** Different fields showing loss of biofilm integrity (× 4k magnification at 15 kV).

FE-SEM analysis of fifth day treated biofilm showed that H-AgNPs were able to eradicate biofilm at MBC concentration (1.8 mg/ml) as shown in **Figure 4**. There was disruption of biofilm as a result of cell lysis caused by H-AgNPs. The nanoparticles were found sticking on the surface of cells (**Figure 5**).

#### Compositional Analysis of Untreated Biofilm by EDS Method

**FIGURE 6:**
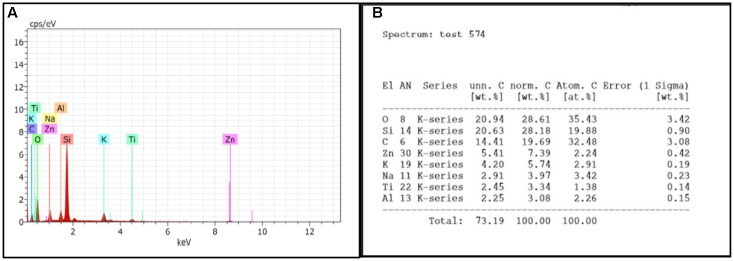
**(A)** EDS graph represents the count per second on *x*-axis and the kilo electron volt which it is generated is on *y*-axis. **(B)** Showing percentage of elements present in control biofilm.

Compositional analysis of control biofilm was performed on fifth day. The results shows the presences of silicon, sodium, potassium, aluminum, carbon, and oxygen. The presence of di-potassium orthophosphate, and sodium chloride in sample was responsible for peak of sodium, potassium, and oxygen whereas peaks of aluminum and silicon were due to support materials, i.e., aluminum stub and glass slide, respectively. The percentage of different elements present in fifth day biofilm are presented in **Figure 6B**.

#### Compositional Analysis of Treated Biofilm by EDS Method

**FIGURE 7:**
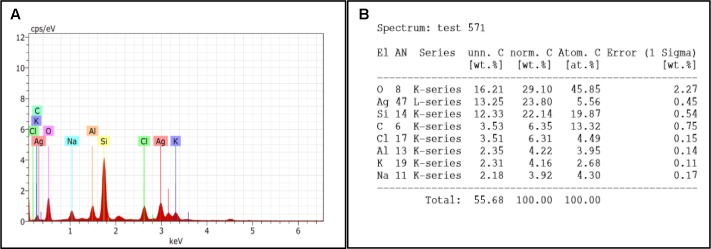
**(A)** EDS graph represents the count second on *x*-axis and the kilo electron volt on which it is generated is on *y*-axis. **(B)** Showing percentage of elements present in treated biofilm.

Fifth day old biofilm treated with H-AgNPs showed two major peaks of silver at 0.1 and 3 KeV along with normal elements which confirmed the presence of silver nanoparticles in the sample. As **Figure 7A** shows the elemental analysis of *K. pneumoniae* biofilm. The percentages of different elements present on fifth day biofilm are presented in **Figure 7B**.

### Synergistic Effect of H-AgNPs and Gentamicin on Biofilm

**FIGURE 8:**
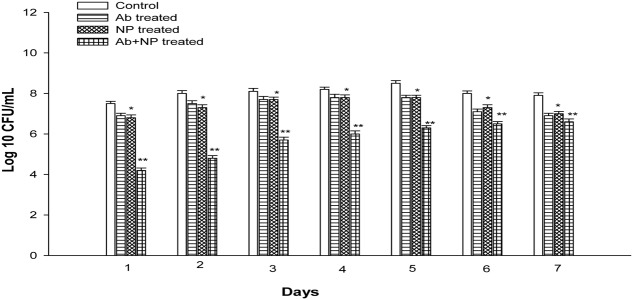
The effect of H-AgNPs alone [fractional inhibitory concentration (FIC)], antibiotic alone (FIC), and H-AgNPs +antibiotic treatment (FIC value) on biofilm. The experiment was performed in duplicate and repeated at least three times on different days. Bars represent standard deviation, ^∗^ denotes (*P* < 0.05) and ^∗∗^ denotes (*P* < 0.01).

The effect of gentamicin and H-AgNPs was studied on *K. pneumoniae* biofilm in combination as well as alone at their FIC value. The results showed that in combination gentamicin and nanoparticles at FIC were quite effective in eradicating biofilm *in vitro* (**Figure 8**). A significant (*P* < 0.01) decrease of 3.3 logs and 3.2 logs was observed on days 1 and 2. A uniform decrease was found on next consecutive days as on peak day, i.e., fifth day 2.2 logs reduction was observed. Similarly, decrease of 1.5 logs and 1.3 logs was observed in the bacterial count on 6 and 7 days. On the contrary gentamicin at FIC alone value showed a decrease of 0.6 logs and 0.5 logs on days 1 and 2. A non-significant decrease of 0.7 logs was observed on peak day (*P* > 0.05). However, gentamicin at FIC alone value was able to reduce biofilm biomass by 0.9 and 1 log on days 6 and 7, respectively. H-AgNPs at FIC alone were also studied for their effect on biofilm. A decrease of 0.7 logs was observed on days 1 and 2 (*P* < 0.05). A decrease of 0.7 logs was observed on peak day also. Moreover, nanoparticles at FIC alone value were able to reduce 0.7 and 0.9 logs on days 6 and 7, respectively, as shown in **Figure 5**. Similarly, semi-quantitative method was performed by CV staining method and the results confirmed decrease in biomass (Supplementary Data).

### Confocal Laser Scanning Microscopy (CLSM) of Cells in Treated and Untreated Biofilm

**FIGURE 9:**
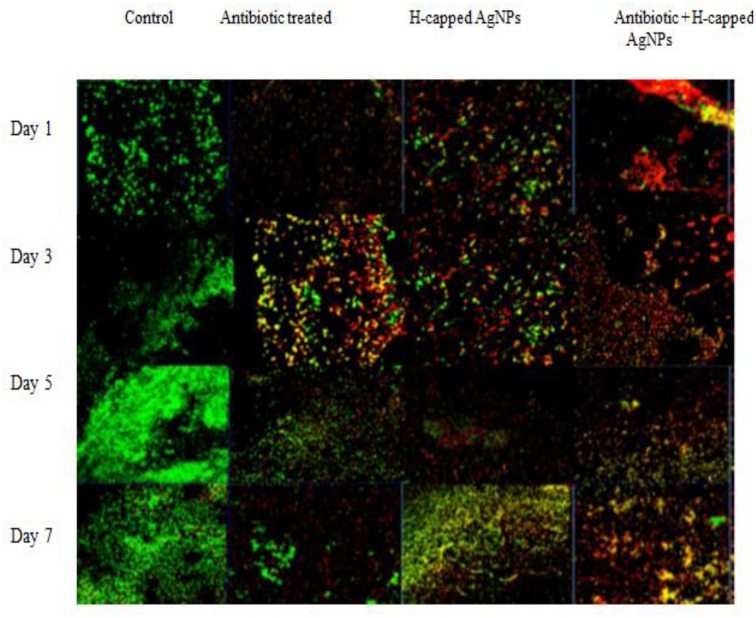
*K. pneumoniae* bioflim stained with LIVE/DEAD Baclight kit and treated with H-AgNPs and gentamicin, alone or in combination. The bioflim observed under CLSM (40x) shows distribution of live/dead bioflim cells on different days of incubation.

*K. pneumoniae* bioflim on coverslips was stained with propidium iodide and SYTO9 dye (LIVE/DEAD Baclight kit) in order to observe the presence of live and dead cells in treated and untreated bioflim. As shown in **Figure 6** (the first column), on defined days as increase in bioflim biomass was observed with time and reached it peak on fifth day. The results also confirmed presence of live cells from days 1 to 5 in large number and their decline in biofilm was observed following tretament with genatmicin and H-AgNPs. The number of live cells was reduced as compared to control biofilm. However, when the agents were applied in combination, a considerable increase in number of dead cells was observed on all days of bioflim treatment (**Figure 9**).

### DNA Fragmentaion Assay

**FIGURE 10:**
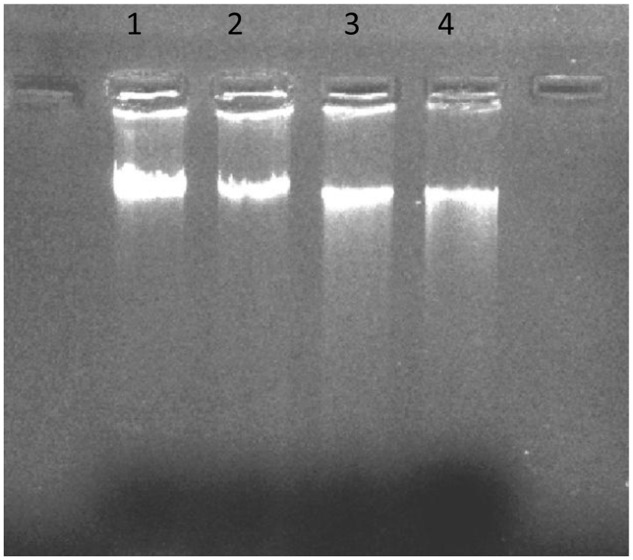
DNA fragmentation following treatment with H-AgNPs. Lane 1 shows DNA control sample of untreated bacterial cells, lane 2 DNA of bacterial pellet treated with the lowest concentration (0.45 mg per ml) of H-AgNPs, lane 3 DNA of bacterial pellet treated with H-AgNPs (0.9 mg per ml), and lane 4 contains the DNA of bacterial pellet treated with H-AgNPs (1.8 mg per ml).

DNA fragmentation assay was performed to check the interaction of cellular DNA and H-AgNPs. *K. pneumoniae* cells were incubated with varying concentartions of H-AgNPs. The results in **Figure 10** shows that there was a linear increase in DNA fragmentation with increase in H-AgNPs concentration. Intact DNA band was seen in lane 1 whereas in lane 2–4, intact DNA concentration was decreased, however, increase in DNA fragmentation was seen with increase in H-AgNPs concentration.

### Cytotoxic Effect of H-AgNPs on THP-1 Cell Line

The results revealed that H-AgNPs showed very low cytotoxic effect on human monocytic immortalized THP-1 cell line. Even at higher dose, i.e., 200 μg/ml, which is higher than the FIC value, H-AgNPs showed negligible toxicity. The results in **Table 1** show that nanoparticles at lower concentartion (10–50 μg/ml) were non-toxic to cells but even at higher concentration (100–200 μg/ml) nanoparticles showed almost negligible cytotoxicity to cells upto 72 h.

**Table 1:** Table showing the cytotoxic effects of H-AgNPs at different concentrations for 24–72 h.

	Average % cell viability of human monocytic immortalized cells THP-1 cell line on incubation with H-AgNPs (*n* = 3)		
Concentration of H-AgNP’s	24 h	48 h	72 h
Control	93.4	92.1	86
10 μg/ml	94.2	93.6	91.5
25 μg/ml	91.9	91.2	90.6
50 μg/ml	92.1	90.5	89.7
100 μg/ml	88.6	85.4	83.3
200 μg/ml	87.9	86.6	81.2

## Discussion

In the present study, an attempt was made to assess the antimicrobial potential of silver nanoparticles functionalized with range of amino acids, which represents an alternative to antibiotic therapy, a novel approach in treatment of bacterial infections particularly those refractive to action of antibiotic. In the initial attempts, H-AgNPs showed significant antimicrobial effect toward a range of standard as well as clinical strains as compared to other amino acid functionalized silver nanoparticles (Kumar et al., manuscript communicated). It is proposed that histidine being a cationic amino acid provides a shield to silver nanoparticles and this results in electrostatic interaction between H-AgNPs and anionic bacterial cell membrane. Histidine rich peptides and glycoproteins have been shown to posses antimicrobial activity against a range of bacterial species (Kacprzyk et al., 2007; Rydengård et al., 2007). The extracellular matrix imparts resistance to the biofilm against various environmental factors including antibiotics (Otto, 2008). Congo red assay revealed that H-AgNPs were capable of reducing extracellular polysaccharide production. To validate our observation H-AgNPs were used for treating *K. pneumoniae* biofilm at MBC concentration for 7 days. In comparison to control, a significant decrease of 3.9 log in bacterial count was found on the peak day of biofilm, i.e., fifth day. These results were confirmed by FE-SEM that revealed changes in the structure of treated as well as untreated biofilm. Though previous researchers have attempted to eradicate biofilm formed by various pathogenic organisms such as ESBL isolates of *E. coli* and *K. pneumoniae* (Ansari et al., 2015), using various strategies but no report is yet available where efficacy of silver nanoparticles alone or in conjunction with antibiotics has been evaluated upto the stage of mature biofilm formation. This is the first report where it is confirmed that green synthesized silver functionalized nanoparticles act as effective antibiofilm agent.

Biofilm age is a major decisive factor in determining the effectiveness of any antibiofilm agent (Sedlacek and Walker, 2007; Ito et al., 2009; Chhibber et al., 2015). As biofilm grows older it becomes difficult to eradicate them by conventional and newer antibiotics due to their altered metabolic activity and thicker extracellular matrix (Rani et al., 2007). Several studies regarding the synergic activity of silver nanoparticles and other agents has been reported (Li et al., 2005; Ruden et al., 2009). An attempt was also made in this study to investigate the potential of H-AgNPs in combination with gentamicin. *In vitro* studies showed that fractional inhibitory concentration index (FICI), i.e., 0.15, of H-AgNPs showed synergic effect when used in conjunction with gentamicin on biofilms (Kumar et al., manuscript communicated). The results showed that both the agents when used at its FIC concentration led to significant 2.2 log reduction in bacterial count. Gentamicin and H-AgNPs alone at FIC value were also used and each showed only 0.7 log reduction in bacterial count on peak day.

Confocal laser scanning microscopy allows the non-destructive study of biofilm making it possible to reconstruct a 3-D structure (Lawrence and Neu, 1999; Psaltis et al., 2007). To further confirm our findings, H-AgNPs alone, gentamicin alone and in combination treated and untreated biofilms were stained using SYTO9 and propidium iodide. The results of confocal microscopy showed enhanced cell killing in combination treatment when compared to treatment with individual agents. Cytotoxicity studies of H-AgNPs on human monocytic immortalized cells THP-1 revealed that nanoparticles were not cytotoxic at even higher doses, i.e., 200 μg/ml. The differential activity of H-AgNPs for bacterial and mammalian cells is attributed to higher negative charge on bacterial membrane than mammalian cells which confirmed that H-AgNPs were promising antibacterial candidate for *in vivo* application. The interaction of H-AgNPs with DNA was also observed. The DNA fragmentation showed that there was a linear increase in DNA fragmentation with increase in H-AgNPs concentration. It is proposed that DNA contains phosphorus as its major components which is known to be soft base and silver as a soft acid reacts with this soft base which leads to destruction of DNA structure. This possibly explains the one of the antimicrobial actions of H-AgNPs as observed in this study.

## Conclusion

The degree of virulence possessed by *K. pneumoniae* and emergence of MDR strains has prompted the scientists to look for newer antibacterial therapeutics. In recent years, various attempts have been made to look for alternative strategies. However, conventional antibiotics and other alternative approaches such as phage therapy, quorum sensing inhibition, phytochemicals are in progress to eradicate the infectious agents but also have their own limitations. Silver nanoparticles despite being potent antimicrobial agent may also confer toxicity to animal cells by means of ion release and oxidative stress at higher concentrations and therefore have *in vivo* administration restrictions which can be addressed in near future. Our study demonstrates the potential of H-AgNPs as promising agent against multidrug resistance strains. This will enable future researchers to mitigate the use of combination of antibiotics and H-AgNPs against multidrug resistant strains.

## Author Contributions

SC: Guidance, designed the experiments and manuscript writing. VG: performed experiments of biofilm and manuscript writing. SS: screening of antibacterial nanoparticles and biofilm analysis. MK: nanoparticle preparation and characterization. RS: provided the lab facilities and guidance for nanoparticles preparation. NW: checked and edited the manuscript.

## Conflict of Interest Statement

The authors declare that the research was conducted in the absence of any commercial or financial relationships that could be construed as a potential conflict of interest. The reviewer TC and handling Editor declared their shared affiliation, and the handling Editor states that the process nevertheless met the standards of a fair and objective review.
