# Supplementary material for: A Novel Approach for Combating Klebsiella pneumoniae Biofilm Using Histidine Functionalized Silver Nanoparticles
Source: Front Microbiol. 2017 Jun 16;8:1104. doi: 10.3389/fmicb.2017.01104 (PMC5472672; doi:10.3389/fmicb.2017.01104)
Supplement: Supplementary file 1 [file Data_Sheet_1.DOCX]

 **Figure 1** Absorbance at 595 nm of *K. pneumoniae* biofilm grown for different days and treated with H-AgNPs.

**Figure 2** Absorbance at 595 nm showing effect of H-capped silver nanoparticles alone (FIC), antibiotic alone (FIC) and H-capped silver nanoparticles+antibiotic treatment (FIC value) on biofilm.
